# STAR3D: a stack-based RNA 3D structural alignment tool

**DOI:** 10.1093/nar/gkv697

**Published:** 2015-07-15

**Authors:** Ping Ge, Shaojie Zhang

**Affiliations:** Department of Electrical Engineering and Computer Science, University of Central Florida, Orlando, FL 32816, USA

## Abstract

The various roles of versatile non-coding RNAs typically require the attainment of complex high-order structures. Therefore, comparing the 3D structures of RNA molecules can yield in-depth understanding of their functional conservation and evolutionary history. Recently, many powerful tools have been developed to align RNA 3D structures. Although some methods rely on both backbone conformations and base pairing interactions, none of them consider the entire hierarchical formation of the RNA secondary structure. One of the major issues is that directly applying the algorithms of matching 2D structures to the 3D coordinates is particularly time-consuming. In this article, we propose a novel RNA 3D structural alignment tool, STAR3D, to take into full account the 2D relations between stacks without the complicated comparison of secondary structures. First, the 3D conserved stacks in the inputs are identified and then combined into a tree-like consensus. Afterward, the loop regions are compared one-to-one in accordance with their relative positions in the consensus tree. The experimental results show that the prediction of STAR3D is more accurate for both non-homologous and homologous RNAs than other state-of-the-art tools with shorter running time.

## INTRODUCTION

Non-coding RNAs (ncRNAs) play diverse cellular functions in biological systems (1–4). Unlike mRNAs whose primary sequences are genetic codes for protein synthesis, the regulatory information of most ncRNAs is encoded in their architectures: the secondary structures defined by the hierarchical assembly of double-stranded stacks and higher-order three-dimensional (3D) structures consisting of packed secondary structure modules interlinked by tertiary interactions (5,6). Therefore, the structural alignments of such ncRNAs can provide essential insight to their functional and evolutionary relationships. However, compared to the development of the computational methods for RNA secondary structure analysis, the progress of RNA 3D structural alignment has been limited. Although the protein 3D structural alignment has been studied for years and many sophisticated methods have been proposed (7–11), it is hard to apply them directly to ncRNAs due to the different properties of their secondary structures.

Recently, with the rapid growth of RNA deposition in the Protein Data Bank (PDB) (12), a number of tools have been developed specifically for the alignments of RNA 3D structures. Generally, they can be categorized into two groups. In the first group, the base pairing interactions in the inputs are ignored or degraded into sequential information. Then the RNAs can be compared using the quadratic-time alignment algorithms. For example, both iPARTS (13) and LaJolla (14) represent RNA backbones as sequences of letters derived from the features of nucleotide torsion angles. iPARTS continues to apply conventional pairwise alignment methods to the encoded linear sequences, while LaJolla searches the similar ‘n-grams’ (substrings of length *n*) in the RNAs by using hash tables. Similar to LaJolla, FRIEs (15) also uses the matching of *k*-mer RNA fragments. In this method, a large set of training fragments from the PDB are clustered into tens of classes based on their structural properties. Each *k*-mer in an RNA can be labeled with the probabilities in these classes, and thus the similarity of two fragments can be measured with the dot product of their probability vectors. Rclick (16) is another RNA 3D structural alignment tool based on the detection of local similarity. The matches between *n*-body cliques (in which *n* member nucleotides satisfy that all pair-wise spatial distances are within a threshold) are determined by the superimposition of their atomic coordinates. With this local structural equivalence, the optimal global alignment is generated by using 3D least squares fitting. Unlike the previously mentioned tools, DIAL (17) incorporates base pairing interactions into its dynamic programming scoring function, which also accounts for sequence and torsion angle information. A penalty is assigned if the pairing attributes (paired or unpaired) of two aligned nucleotides are different. Elastic Shape Analysis (ESA) models the RNA 3D structures not as sequences but as curves in a four-dimensional space: the atomic coordinates are in 3D space and the sequence information is encoded as an additional dimension (18). Then the similarity between two RNAs can be evaluated by minimizing their geodesic distance with a quadratic-time dynamic programming algorithm.

The other group of RNA 3D structural alignment tools relies on the comparison of base pairing interactions in the molecules. In ARTS (19), two successive base pairs are used together as a seed. The optimal matching of seeds in two RNAs is extended globally to the unpaired regions and the result is refined with the least squares fitting technique. Similar to ARTS, the final results of R3D Align (20) are assembled from the alignments of local neighborhoods. Neighbors are the spatially closest nucleotides in one RNA, which may imply interactions such as base pairs, tertiary interlinks and stacking contacts. The structurally similar neighbors in two RNAs are detected and the optimal combination of these local alignments is determined by employing maximum clique finding algorithm on a compatibility graph. SARA (21) does not discriminate the paired and unpaired regions in RNAs. Inspired by a protein 3D structural alignment method named MAMMOTH (22), SARA describes the backbone of an RNA as a series of unit-vectors. The distances between the unit spheres of inputs can be measured with URMS (unit-vector root mean square) and the corresponding global alignment is identified by using dynamic programming. The same procedure is applied only to base pairs if the pairing information is provided. The 3D structural alignment of entire RNAs can be optimized based on the mapping of pairing interactions. SETTER (23) integrates stacks and loops into the RNA 3D structural alignment method. It splits the RNA sequences into GSSUs (generalized secondary structure units), each of which has a loop, a neck and a stem. The highly similar GSSU pairs are used as seeds to guide the alignment of other GSSUs. To simplify the computation, the exact mapping of nucleotides is ignored in this method.

It can be seen that the RNA secondary structural information, in particular the hierarchical topology of stacks, is not used in the reviewed methods. However, the enclosing and juxtaposing relations between stacks provide more detailed structural information than what has been used in the existing tools, such as ‘paired’ or ‘unpaired’ attributes, base pairing interactions and stack positions. The issue is the difficulty of integrating the conventional RNA secondary structure alignment algorithms into the RNA 3D structural comparison. Given the high time complexity of these algorithms [at least *O*(*n*^3^)] (24–27), applying them directly to the relatively complicated atomic coordinates will increase the computational complexity significantly.

Here, we propose a novel RNA 3D structural alignment tool called STAR3D that explicitly makes use of the conservation of secondary structures with high efficiency. It aims at finding the consensus of stacks by using 2D topology and 3D geometry first, and then uses it to guide the alignments of the loop regions. To achieve this goal, first, the sub-stacks with similar 3D structures are detected and assembled into conserved stack pairs. Then, a compatible graph is constructed based on their secondary structural relations and spatial distances. In this graph, the maximum clique can be converted into a tree-like consensus structure of two RNAs. After that, the loop regions are ordered by the common tree. Each of them only needs to be compared with its partner by using 3D information. STAR3D has been implemented in Java. The benchmarking results show that STAR3D outperforms the state-of-the-art RNA 3D structural alignment tools with high efficiency.

## MATERIALS AND METHODS

### Preprocessing

The inputs of STAR3D are the atomic coordinates of two polymer RNA chains, which are presented in the corresponding PDB files. They are preprocessed to obtain the RNA secondary structures to guide the 3D structural alignment. All plausible pairing interactions are identified by using MC-Annotate (28,29). Among them, the Watson–Crick base pairs (A↔U, C↔G) and wobble base pairs (G↔U) are retrieved to form the RNA secondary structures (30). Other pairing interactions are considered during the loop alignment. In order to avoid excessive computation, we eliminate the crossing base pairs in the secondary structures by using the program RemovePseudoknots (31) in the RNAstructure package (32). The discarded stems are used as pairing interactions in the loops.

### Stack decomposition

Helical structured stacks are formed by consecutively nested Watson–Crick base pairs and wobble base pairs. To detect the 3D structural conservation in the stacks efficiently, the double-stranded regions in the pseudoknot-free secondary structures are decomposed into consecutive sub-stacks of size *k*, namely *k*-stacks. A stack with *l* base pairs (*l* ≥ *k*) can be divided into *l* − *k* + 1 overlapping *k*-stacks. All the possible *k*-stacks are collected for further processing.

Based on the definition of *k*-stack, we introduce some basic notations. Given an RNA *A*, the 3D coordinates of the *i*-th residue are denoted as *A*[*i*]. At the secondary structure level, the set of *k*-stacks in the pseudoknot-free structure is denoted as $\mathcal {P}^A$. For a specific *k*-stack $p^A \in \mathcal {P}^A$, the index of the leftmost base (5′ end) is represented as *b*(*p*^*A*^) and the index of the rightmost base (3′ end) is represented as *e*(*p*^*A*^). Thus the 3D coordinates of the double-stranded subsequences in *p*^*A*^ are *A*[*b*(*p*^*A*^)…*b*(*p*^*A*^) + *k* − 1] and *A*[*e*(*p*^*A*^) − *k* + 1…*e*(*p*^*A*^)], which are defined as 3*D*(*p*^*A*^).

### Detecting the conserved stack regions

**Figure 1.**
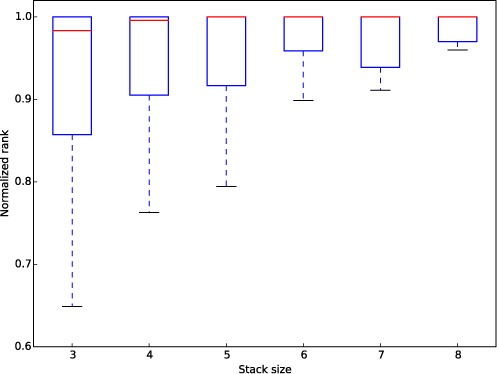
The normalized ranks of the matched stacks in two 23S rRNAs (PDB 2j01, chain A and PDB 2aw4, chain B). The structure similarity is measured with RMSD. For a stack of size k in 2j01, its RMSDs to all the size-k stacks in 2aw4 are computed.

STAR3D identifies the stack components conserved in 3D structures as anchors and uses them to constrain the global alignment. Similar approaches have been applied in numerous computation-efficient tools for genome alignment (33,34) and RNA secondary structure alignment (35,36). The difference is that STAR3D uses the 3D coordinates of atoms to detect the potential homologous regions. Given the fact that RNA stacks adopt an A-form helical conformation, a major issue needs to be addressed: whether the 3D structural similarity of conserved stack regions is significant enough to distinguish them from the random ones. To answer the question, we have conducted a statistical research on the matched stacks in a hand-crafted alignment (37). The survey results in Figure 1 indicate that the orthologous sub-stacks have highly similar 3D structures, and they can be detected by evaluating RMSD. In our method, the *k*-stacks (default value of *k* is 3) in the inputs are retrieved as the building blocks for the larger conserved regions. Shorter helices are not considered because of their low occurrence in the real RNAs.

Given two input RNAs *A* and *B*, the two sets of *k*-stacks $\mathcal {P}^A$ and $\mathcal {P}^B$ are sorted in ascending order by the leftmost bases. The three-dimensionally conserved *k*-stacks in *A* and *B* are determined by their RMSDs. *C*_*i*, *j*_, the indicator of conservation for $p^A_i$ and $p^B_j$, is computed using the following function:

(1) \begin{equation*} C_{i, j}=\left\lbrace \begin{array}{l l}1 & \quad RMSD(3{\rm D}(p^A_i), 3{\rm D}(p^B_j))<r_{\rm c}\\ 0 & \quad \mbox{otherwise} \end{array} \right. \end{equation*}

**Figure 2.**
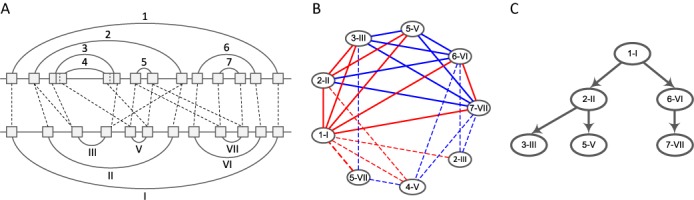
A description of the basic data structures used in STAR3D. (**A**) The *e*-stack pairs in two artificial RNAs. The gray boxes show the *e*-stacks and the dashed lines show the matching between them. *e*-stack 3 and *e*-stack 4 are overlapped with each other. (**B**) The compatible graph of *e*-stack pairs in (A). Red color marks the enclosing relations and blue color marks the juxtaposing relations. The solid lines show the edges in the maximum clique. To simplify the presentation, the 3D similarity requirement is not considered in the figure. (**C**) The tree-like consensus of *e*-stacks obtained from the clique in (B).

The function *RMSD* measures the average spatial distance between the superimposed residues in $3{\rm D}(p^A_i)$ and $3{\rm D}(p^B_j)$, with *r*_c_ being the RMSD cutoff (default value is 4 Å) (21). In our implementation, the RMSD values are computed with the Kabsch method (38) by using the geometric center of six backbone atoms C3’, C4’, C5’, O3’, O5’ and P (39). The indicators for all pairs of *k*-stacks ($\mathcal {P}^A \times \mathcal {P}^B$) are stored in a matrix. Then, we extend the consecutive matches of *k*-stacks to form larger ungapped alignments. For instance, $p^A_i, p^A_{i+1}$ and $p^B_j, p^B_{j+1}$ can be merged into two aligned stacks of size *k* + 1, if *C*_*i*, *j*_ = *C*_*i* + 1, *j* + 1_ = 1, $b(p^A_i)=b(p^A_{i+1})-1$, $e(p^A_i)=e(p^A_{i+1})+1$, $b(p^B_j)=b(p^B_{j+1})-1$ and $e(p^B_j)=e(p^B_{j+1})+1$. This procedure continues through the diagonals of the matrix until all the constructed alignments can not be extended any further. The two stack components in an assembled alignment are called extended stacks, written shortly as *e*-stacks. Correspondingly, the aligned *e*-stacks form *e*-stack pairs. We define the sets of *e*-stacks in *A* and *B* as $\mathcal {Q}^A$ and $\mathcal {Q}^B$, and the set of *e*-stack pairs as $\mathcal {S}$. According to the definition of *e*-stack, the cardinalities of $\mathcal {Q}^A$, $\mathcal {Q}^B$ and $\mathcal {S}$ are identical. As a result, we denote the members of a specific *e*-stack pair *s*_*i*_($\in \mathcal {S}$) as $q^A_i$($\in \mathcal {Q}^A$) and $q^B_i$($\in \mathcal {Q}^B$) ($s_i=(q_i^A, q_i^B)$). Note that *e*-stacks may overlap with each other [see Figure 2A]. Unlike *k*-stacks, the sizes of *e*-stacks are not fixed. Therefore, we define a new notation *l*(*q*^*A*^) to represent the number of base pairs in *q*^*A*^. Hence 3*D*(*q*^*A*^) are *A*[*b*(*q*^*A*^)…*b*(*q*^*A*^) + *l*(*q*^*A*^) − 1] and *A*[*e*(*q*^*A*^) − *l*(*q*^*A*^) + 1…*e*(*q*^*A*^)].

For some large RNAs, the numbers of *e*-stack pairs are too large for computation. To determine the highly significant ones, we consider two criteria: the RMSD between two *e*-stacks and their size. The significant scores of *e*-stack pairs are defined using the formula $RMSD(3D(q^A_i), 3D(q^B_i))-0.1 \times l(q^A_i)$. They are sorted in ascending order and only 200 top-ranked pairs are retained for further processing. Based on our study, 200 high-scoring *e*-stack pairs are sufficient to cover most of the conserved helical regions in 23S rRNAs, the largest RNAs in PDB. More *e*-stack pairs may be used by setting the parameter if more complex structures are presented.

### Assembling the consensus of stacks

To generate a consensus of stacks, the positions of *e*-stack pairs in the secondary structures and 3D space are analyzed. In the pseudoknot-free secondary structure of *A*, two *e*-stacks $q^A_i$ and $q^A_j$ may have one of the three following relations: (i) $q^A_i$ and $q^A_j$ are overlapping (denoted by $q^A_i\otimes q^A_j$); (ii) $q^A_i$ encloses $q^A_j$ (denoted by $q^A_i \prec _E q^A_j$); (iii) $q^A_i$ is before and juxtaposed to $q^A_j$ (denoted by $q^A_i \prec _J q^A_j$). In our algorithm, $q^A_i$ *directly* encloses $q^A_j$ if $q^A_i \prec _E q^A_j$ and $\nexists k (q^A_i \prec _E q^A_k \prec _E q^A_j)$ (denoted by $q^A_i <_E q^A_j$). Similarly, we say $q^A_i$ is *directly* before and juxtaposed to $q^A_j$ if $q^A_i \prec _J q^A_j$ and $\nexists k (q^A_i \prec _J q^A_k \prec _J q^A_j)$ (denoted by $q^A_i <_J q^A_j$).

Notice that both ≺_*E*_ and ≺_*J*_ are strict partial orders, so the non-overlapping *e*-stacks in an RNA can form a directed acyclic graph. It is well-known that the RNA secondary structures have a tree-like topology (40–43). Thus we model the non-overlapping relations of *e*-stacks in *A* as a tree:

- Assign a pseudo stack $q_{\bullet }^{A}$ ($b(q_{\bullet }^{A})=0, e(q_{\bullet }^{A})=|A|+1, l(q_{\bullet }^{A})=0$) to the root node.
- Connect $q_i^{A}$ to $q_j^{A}$ if $q_i^{A} <_E q_j^{A}$.
- Order the children nodes of $q_i^{A}$ in ascending order based on ≺_*J*_.

We also define the compatible *e*-stack pairs: *s*_*i*_ and *s*_*j*_ are compatible if $(q^A_i,q^A_j) \in R$, $(q^B_i, q^B_j) \in R$, and *R* ∈ {≺_*E*_, ≻_*E*_, ≺_*J*_, ≻_*J*_}. The non-compatible *e*-stack pairs can not be in the consensus together because their members are disordered in the secondary structures.

Furthermore, we can prove the following lemma:

#### Lemma 1

*For a non-empty set* $\mathcal {S^{\prime }} \subseteq \mathcal {S}$, *if any two of* *e*-*stack* *pairs* $s_{I}=(q^A_i, q^B_i)$ *and* $s_{j}=(q^A_j, q^B_j) \in \mathcal {S^{\prime }}$ *are compatible, the corresponding two e-stack sets have the same tree structure*.

#### PROOF

Without loss of generality, we assume that $q^A_i$ is a child of $q^A_{\bullet }$ and $q^B_i$ is not a child of $q^B_{\bullet }$. Then $q^B_i$ must be on a subtree rooted at one child of $q^B_{\bullet }$. Thus at least two stacks, $q^B_{\bullet }$ and $q^B_j$, enclose $q^B_i$. However $q^A_i$ has only one ancestor $q^A_{\bullet }$. It is a contradiction to the conditions, because *s*_*i*_ and *s*_*j*_ are not compatible. So the children of $q^A_{\bullet }$ and $q^B_{\bullet }$ are from the same set of *e*-stack pairs. Based on Step (iii) of the tree construction procedure, the orders of their children should be the same. Then it is proved that the lemma holds for the top two levels of the trees. Assume it is also true for the top *n* levels. Then for an *e*-stack $q^A_{i^{\prime }}$ at *n*-th level, its partner $q^B_{i^{\prime }}$ must also be at *n*-th level and their relative positions on the trees are the same. Assume an *e*-stack $q^A_{j^{\prime }}$ is a child of $q^A_{i^{\prime }}$ and $q^B_{j^{\prime }}$ is not a child of $q^B_{i^{\prime }}$. First $q^B_{j^{\prime }}$ must be on a subtree rooted at $q^B_{i^{\prime }}$. Otherwise $s_{i^{\prime }}$ and $s_{j^{\prime }}$ are not compatible. Second, $q^B_{j^{\prime }}$ can not be at (*n* + 2)-th or lower levels, otherwise the numbers of the ancestors of $q^A_{j^{\prime }}$ and $q^B_{j^{\prime }}$ are different, which is a contradiction to the conditions. So the children of $q^A_{i^{\prime }}$ and $q^B_{i^{\prime }}$ are also from the same set of *e*-stack pairs, and they can be sorted by the juxtaposing relation. By induction, we know the lemma is true.

Lemma 1 indicates how to find the 3D structural consensus of the stack regions in two input RNAs. Thus, to detect the *e*-stack configuration for the consensus, we construct a compatible graph. The vertices are the *e*-stack pairs. A vertex *s*_*i*_ is connected to another one *s*_*j*_ if they meet two requirements. First, *s*_*i*_ and *s*_*j*_ are compatible, which ensures the *e*-stacks in them are well-ordered in the secondary structures. Second, *s*_*i*_ and *s*_*j*_ must satisfy $RMSD(3D(q^A_i) \circ 3D(q^A_j), 3D(q^B_i) \circ 3D(q^B_j)) < r_c$, which implies that *s*_*i*_ and *s*_*j*_ share similar rigid transformation (‘$\circ$’ means the concatenation operation which joins the lists of 3D coordinates end-to-end). Based on the graph properties, the optimal stack configuration can be inferred from the maximum clique in the graph, which is detected by using the Bron-Kerbosch algorithm (44). After that, the 3D structural alignment in the double-stranded regions is determined by the topology of these vertices in the clique. Note that the *e*-stacks are the maximal 3D conversed regions in the helices (they can not be extended any more). Therefore, the 3D similarity requirement will filter most of the improper edges, and make the compatible graph very sparse. Although normally finding the maximum clique takes exponential time, it is solved very efficiently in our method. Figure 2B and C show a compatible graph and the corresponding consensus of stacks. The detected consensus is the ‘core’ of the 3D structural conservation and it will work as an anchor for the following loop alignment. The double-stranded regions not in the consensus, such as stack 4 in Figure 2A, are considered as loops in the following computation. The corresponding Watson–Crick base pairs and wobble base pairs are also used as interactions in the loop regions to assist the alignment.

### Loop alignment using 3D information

With the tree-like consensus of stacks, all the other regions not in it can be divided into ordered loops. For one leaf node, two hairpin loops enclosed in two *e*-stacks can be identified. For the internal nodes, their enclosed regions are split by their children nodes into internal loops, bulges, or multi-loops. Hence, the numbers of loops in the inputs are the same, and we can find the mapping of them by traversing the tree. This approach has two benefits. First, the computational efficiency of loop alignment can be improved significantly for large RNAs, because only the matched loops need to be aligned together. Second, the superimposition of stack regions can be used to guide the 3D structural alignment of loop regions. For the functional RNAs, the stack regions are more conserved than the loop regions. Thus, any RMSD computation during the loop alignment uses the rotation and translation of the stack alignment.

A dynamic programming algorithm with quadratic-time complexity is applied to the 3D structural alignment of two loops. Assume the 3D structures of *k*-th pair of matched loops are $A[i_k \ldots i_k+n_{k_1}-1]$ and $B[j_k \ldots j_k+n_{k_2}-1]$. To simplify the description and computation, we denote them as $L^A_k[1 \ldots n_{k_1}]$ and $L^B_k[1 \ldots n_{k_2}]$, whose starting index is 1. Thus, the recursive function is ($1 \le i \le n_{k_1}$, $1 \le j \le n_{k_2}$):

(2) \begin{equation*} \begin{aligned} I_{i,j} & =\max \lbrace M_{i-1, j}+\epsilon _o+\epsilon _e, I_{i-1, j}+\epsilon _e, D_{i-1, j}+\epsilon _o+\epsilon _e\rbrace \\ D_{i,j} & =\max \lbrace M_{i, j-1}+\epsilon _o+\epsilon _e, I_{i, j-1}+\epsilon _o+\epsilon _e, D_{i ,j-1}+\epsilon _e\rbrace \\ M_{i,j} & =\max \lbrace I_{i-1,j-1}, D_{i-1,j-1}, M_{i-1, j-1}\rbrace +\alpha (i, j)+ \beta (i,j) \end{aligned} \end{equation*}

Here, ε_*o*_ and ε_*e*_ are the gap open penalty and gap extension penalty. *I*, *D*, *M* denote the optimal alignment scores for insertions, deletions and substitutions, respectively. These functions are initialized with *M*_0, 0_ = *I*_0, 0_ = *D*_0, 0_ = 0, *M*_*i*, 0_ = *M*_0, *j*_ = −∞, *I*_*i*, 0_ = ε_*o*_ + ε_*e*_ × *i*, *D*_0, *j*_ = ε_*o*_ + ε_*e*_ × *j*, *I*_0, *j*_ = *D*_*i*, 0_ = −∞. The optimal score is $\max (I_{n_{k_1}, n_{k_2}}, D_{n_{k_1}, n_{k_2}}, M_{n_{k_1}, n_{k_2}})$ and the exact 3D structural alignment for the two loops can be found by using traceback.

The scores for substitution contain two parts: α(*i*, *j*) and β(*i*, *j*). The function α(*i*, *j*) is based on the 3D distance between two bases. The corresponding formula is:

(3) \begin{equation*} \alpha (i,j)=\left\lbrace \begin{array}{l l}-\infty & d_{i,j} \ge 2 \cdot r_c \\ {\rm mismatch}\_{\rm score} & 2 \cdot r_c >d_{i,j} \ge r_c \\ 0.5 \times {\rm match}\_{\rm score} & r_c > d_{i,j} \ge 0.5 \cdot r_c \\ {\rm match}\_{\rm score} & 0.5 \cdot r_c > d_{i,j} \\ \end{array} \right. \end{equation*}

where *d*_*i*, *j*_ denotes the RMSD between two nucleotides $L^A_k[i]$ and $L^B_k[j]$. Note that they are superimposed with the transformation of aligned stack regions. To capture the backbone conformation, STAR3D uses 3-nt regions, $L^A_k[i-1, i, i+1]$ for $L^A_k[i]$ and $L^B_k[j-1, j, j+1]$ for $L^B_k[j]$, in the computation of *d*_*i*, *j*_. The possible values of *d*_*i*, *j*_ can be categorized into three groups. The two nucleotides are not allowed to be aligned if the spatial distance is too large (≥2 · *r*_c_). Otherwise, they are defined to be ‘matched’ or ‘mismatched’ and the matched nucleotides may be assigned with two different scores.

The second function β(*i*, *j*) calculates the bonus scores for the base pairs in loop regions. Pseudoknots, non-canonical base pairs and canonical base pairs in the unaligned stack regions are considered in the computation. Due to the potential crossing in pseudoknots and non-canonical base pairs, finding the optimal matching of these pairing interactions is an NP-hard problem. To reduce the running time, we propose a heuristic algorithm to solve the problem. Generally, each base has three possible pairs: Watson–Crick base pair, Hoogsteen base pair and Sugar base pair (45). All the predicted base pairs of two nucleotides $L^A_k[i]$ and $L^B_k[j]$ are compared in 3D space by using a similar approach of comparing nucleotides in α(*i*, *j*). The match of two base pairs is valid if the corresponding RMSD is less than *r*_c_. The maximum number of matched pairs is returned as the result of β(*i*, *j*). Thus the problem is converted into bipartite graph matching, which can be solved by dynamic programming.

## RESULTS

### Benchmarking tools

STAR3D is benchmarked with ARTS, LaJolla (v2.2), SARA (v1.0.7) and R3D Align in this section. Their batch programs are available and widely used for performance testing. In addition, they can output the exact one-to-one mapping of nucleotides, which is important for the analyzing of specific alignments of homologous and non-homologous RNAs. R3D Align is dedicated to homologous RNAs. To make the comparison fair, it is only used in the experiments for homologous rRNAs. An in-house modification of LaJolla is implemented to output not only the rigid transformation but also the exact alignments. All the experimental results were performed with default parameters. To evaluate the secondary structure similarity and optimize the superimposition, ‘-b’ and ‘-s’ options are specified for SARA. Both ARTS and LaJolla generate ‘disordered alignments’, e.g. *a*_*i*_ is aligned to *b*_*j*_, *a*_*k*_ is aligned to *b*_*l*_, while *i* < *k* and *j* > *l*. For ARTS, the largest proper alignment is retrieved; for LaJolla, the improper alignment is discarded since only one result is returned from the modified implementation.

### Alignment quality assessment with R-FSCOR dataset

The R-FSCOR dataset (46) contains 192 chains collected from the SCOR database (47). In SCOR, the chains with at least three base pairs and unique function annotations are clustered at 90% identity. The representative in each cluster is selected into the R-FSCOR dataset. The performance of four tools is compared by calculating PSI (percentage of structural identity) and PSS (percentage of aligned secondary structure) values of the all-to-all alignments for the R-FSCOR dataset. PSI is defined as the percentage of aligned nucleotides in 4 Å with respect to the length of the shorter sequence. PSS is defined as the percentage of aligned base pairs in 4 Å with respect to the smaller number of base pairs of two aligned RNA sequences. PSI and PSS have been used as replacement for RMSD to evaluate the quality of the 3D structural alignment (21,23). The base pairs in the tested chains, including both canonical base pairs and non-canonical base pairs, are predicted using MC-Annotate. All programs in this experiment were executed on a CentOS cluster with 100 nodes.

**Figure 3.**
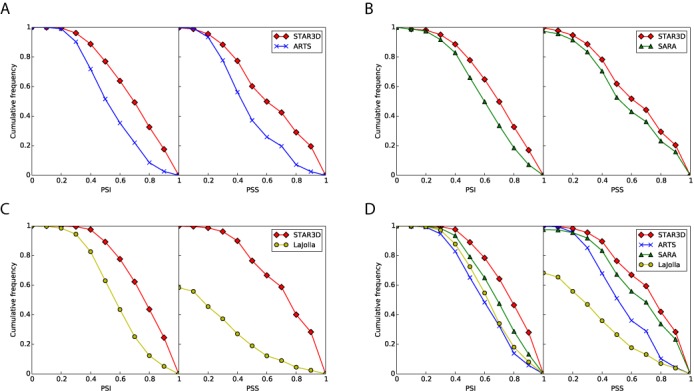
The cumulative frequencies of the PSI and PSS values of STAR3D, ARTS, SARA and LaJolla in different experiments. (**A**) STAR3D versus ARTS. (**B**) STAR3D versus SARA. (**C**) STAR3D versus LaJolla. (**D**) All four tools.

None of the tools can find alignments for all the inputs. ARTS outputs 11 385 proper alignments, LaJolla outputs 7771 proper alignment, SARA outputs 18 335 alignments and STAR3D outputs 17 455 alignments, respectively. For STAR3D, no alignment is generated if the sizes of all potential *e*-stacks in the inputs are less than *k*(=3). However, the alignments for these inputs can be detected if a smaller *k* (e.g. 2) is specified. In addition, RNAMotifScanX (48), which is also designed by our lab for searching RNA 3D structural motifs in the single-stranded regions, can be applied since those RNAs are relatively short and are dominated by loops. STAR3D was compared with ARTS, SARA and LaJolla one by one. To make the comparison fair, the inputs are not considered if STAR3D or the corresponding benchmarking tool can not generate alignments for them. Table 1 summarizes the mean PSI and PSS values of four tools in the experiments. It can be seen that STAR3D outperforms the other three tools by a large margin: the PSIs are increased by 13 to 30% and the PSSs are increased by 10 to 190%. The low PSS values of LaJolla may be caused by ignoring of the secondary structural features. ARTS and SARA have relatively high PSS values because the base pairing information is integrated. For SARA, the optimization step after the backbone alignment may contribute to its better performance than ARTS. By considering the secondary structures of two input RNAs, STAR3D accurately predicts the matching of the stack regions, which is demonstrated by the high PSS values. And guided by the consensus of stacks, STAR3D provides best global alignments in all four tools without an optimization step, which is shown by the high PSI values. We also find that the running time of STAR3D for the whole procedure is much shorter (at least 1/10) than the other three tools. A detailed discussion about the computational efficiency will be shown in a later section. Figure 3 shows the cumulative frequencies of the PSI and PSS values in different comparisons. Figure 3D is based on the valid inputs for all tools. It can be seen that some alignments of SARA and LaJolla may not contain any base pair. On the other hand, the PSS values of ARTS and STAR3D are all greater than zero, because ARTS extends the base pair mapping and STAR3D relies on the stack mapping. What's more, from Figure 3D, we can see that PSI curves between 0.0 and 0.2 are very similar for all tools. The major performance difference between STAR3D and the other three tools is at the range from 0.4 to 0.7, which indicates STAR3D may be more sensitive to the local conservation of RNAs.

**Table 1.** The comparison of mean PSI and PSS values between STAR3D and three other tools by using the R-FSCOR dataset

	# of overlapped alignments	ARTS		SARA		LaJolla		STAR3D	
		PSI	PSS	PSI	PSS	PSI	PSS	PSI	PSS
ARTS versus STAR3D	11 054	0.538	0.485					**0.682**	**0.632**
SARA versus STAR3D	17 454			0.601	0.580			**0.679**	**0.638**
LaJolla versus STAR3D	7397					0.580	0.251	**0.754**	**0.729**
Consensus	4451	0.600	0.549	0.683	0.668	0.627	0.318	**0.764**	**0.729**

The total number of inputs is 18 336. ARTS, SARA, LaJolla and STAR3D output 11 385, 18 335 , 7771 and 17455 alignments, respectively. Best performance is set to bold.

**Table 2.** Running time (in seconds) of ARTS, LaJolla, SARA, R3D Align and STAR3D for the homologous alignments of 16S and 23S rRNAs

rRNAs	ARTS	LaJolla	SARA	R3D Align	STAR3D
*H. marismortui* and *E. coli* 23S	117.2	119 886.7	27 035.2	751.7	**1.7**
*H. marismortui* and *T. thermophilus* 23S	98.5	125 835.9	26 184.8	573.4	**2.0**
*E. coli* and *T. thermophilus* 23S	79.7	152 635.6	27 467.3	653.2	**1.4**
*E. coli* and *T. thermophilus* 16S	20.1	16 209.3	4714.4	308.9	**1.1**

Best performance is set to bold. The preprocessing time is not included for ARTS, SARA, R3D Align, and STAR3D.

### Structural alignments of non-homologous RNAs

Identifying the conserved regions in non-homologous RNAs is a major aim of the RNA 3D structural alignment tools. In this section, we will analyze the different strategies of STAR3D and three other tools by showing the alignments of non-homologous RNAs. The RNAs in the examples are obtained from the R-FSCOR dataset.

**Figure 4.**
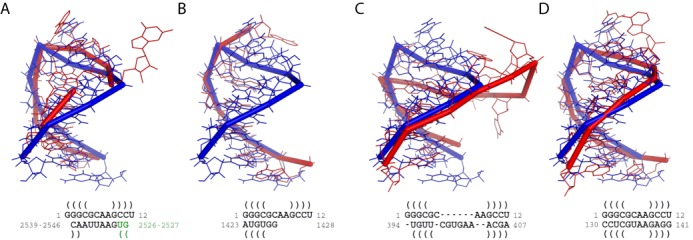
The alignment results for the GNRA motif (PDB: 1zih, chain A) and the 23S rRNA (PDB: 1njo, chain 0). (**A**) The result of LaJolla. (**B**) The result of SARA. (**C**) The result of ARTS. (**D**) The result of STAR3D. The blue ribbons show the 3D structure of the GNRA motif and the red ribbons show the 3D structures of the aligned regions in the 23S rRNA. The secondary structural alignments are listed below the 3D structure figures and the base pairs are predicted by MC-Annotate. The green letters in the LaJolla alignment mark the disordered nucleotides (2526–2527).

The first example is the alignment between a GNRA motif (PDB 1zih, chain A) and a *Deinococcus radiodurans* (*D. radiodurans*) 23S rRNA (PDB 1njo, chain 0). The aligned regions and the corresponding secondary structures are shown in Figure 4. Although it has a decent stack mapping (residue 2526 is paired with residue 2540 and residue 2527 is paired with residue 2539), the alignment produced by LaJolla is disordered: residue 2526–2527 should be at the 5′ side of residue 2539. For SARA, the aligned region of the rRNA is highly conserved with a segment of the motif. However, only one strand of the motif is aligned and the corresponding loop regions are very different. One possible reason is that the unit-vectors used by SARA only describe the conformation of the backbone. Furthermore, the alignments of base pairs and the whole 3D structures are computed separately. Thus it may overlook the pairing information if the partial structure alignment achieves the maximum score. ARTS finds the matching of base pairs first and then extends it to both 5′ and 3′ directions of the RNA strand. In Figure 4C, it can be seen that three base pairs are matched very well, while the 3D structures of the loop regions are distinct. This may be caused by the different treatment of stacks and the corresponding loops in the computation of ARTS. For STAR3D, the entire motif (residue 1–12) is aligned to the residues 130–141 in the rRNA. The tetraloop of the rRNA has the common structural characteristics of the GNRA motif: the four residues are ‘GUAA’ and the loop is closed by a ‘C↔G’ pair. The 3D structural alignment in Figure 4D also shows that the detected region in the 23S rRNA has a very high probability to be a GNRA motif. Similarly to the strategy of ARTS, the conserved stack regions are detected first in STAR3D. However, STAR3D ensures that the corresponding loops should have similar rigid transformation with the stacks, otherwise, the entire alignment will be assigned a low score.

**Figure 5.**
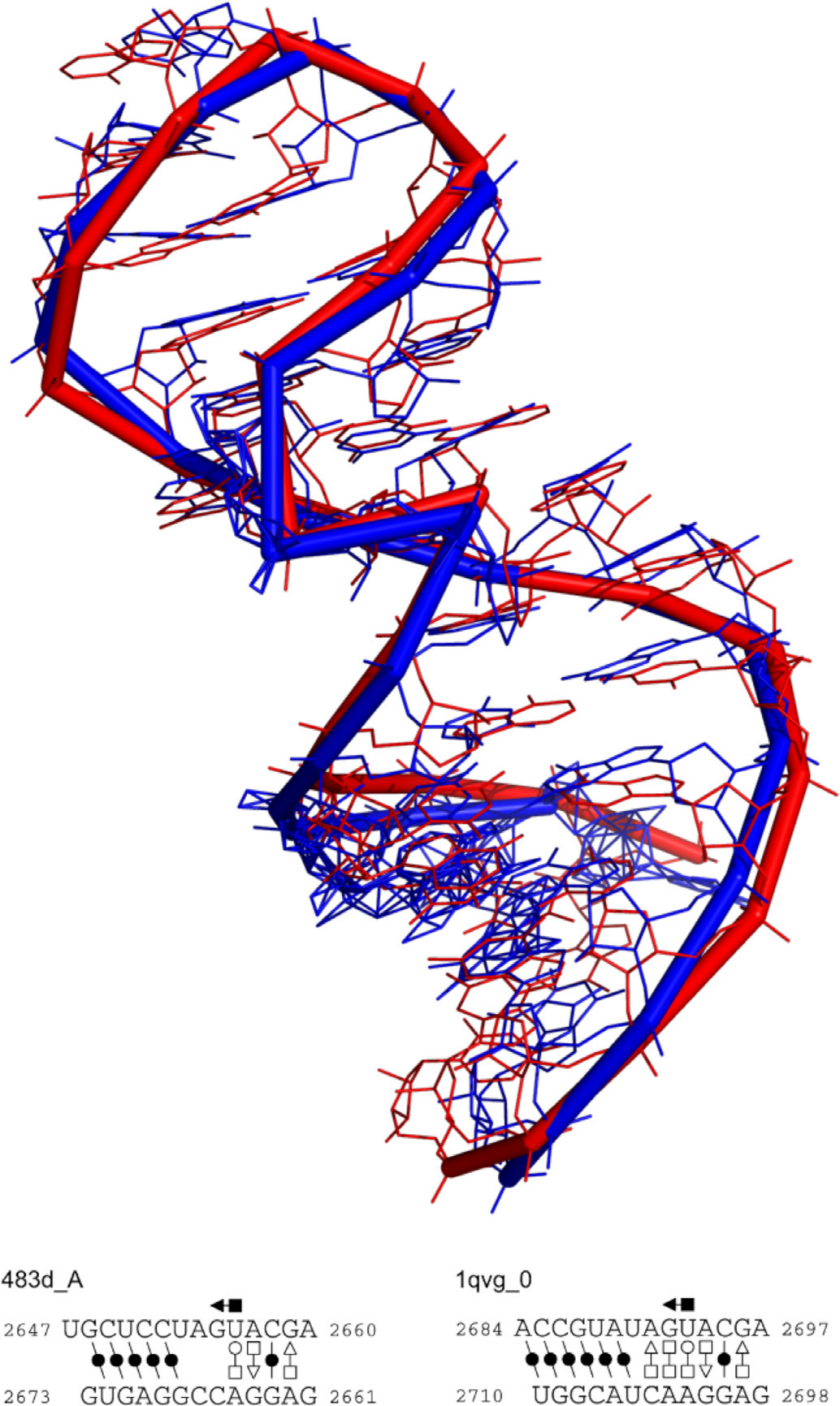
The alignment result of STAR3D for the sarcin-ricin motif (PDB: 483d, chain A) and the 23S rRNA (PDB: 1qvg, chain 0). The blue ribbon shows the 3D structure of the sarcin-ricin motif and the red ribbon shows the 3D structure of the 23S rRNA. The base pairs in the aligned regions are predicted by MC-Annotate.

The sarcin-ricin motif is an important structural motif involved in the interaction between rRNAs and the elongation factors (49). In the R-FSCOR dataset, there is one chain of 23S sarcin-ricin motif (PDB: 483d, chain A) and 22 23S rRNAs, 11 from *Haloarcula marismortui* (*H. marismortui*) and 11 from *D. radiodurans*. The 3D structural alignments of the motif and all the 23S rRNAs are analyzed. Compared with the GNRA motif, sarcin-ricin is more complex: it contains 27 residues, 6 canonical base pairs and 4 non-canonical base pairs. For ARTS and LaJolla, no highly conserved region is found in those rRNAs. SARA can detect potential motifs in all the *H. marismortui* 23S rRNAs, but none in the *D. radiodurans* 23S rRNAs. STAR3D not only finds the motif candidates in *H. marismortui* 23S rRNAs, but also in 6 of 11 *D. radiodurans* 23S rRNAs. To verify the detected motifs, the docking results of the alignments are analyzed. An example alignment of the sarcin-ricin motif and one *H. marismortui* 23S rRNA is shown in Figure 5. From the base pair profiles and the 3D structures, it can be seen that the hairpin loop (residues 2684–2710) has a high probability to be a sarcin-ricin motif. By checking the base pair annotation of all the *D. radiodurans* 23S rRNA, we can find the structural variance in the motif regions. For the five rRNAs in which STAR3D can not detect the motifs, only four base pairs are annotated in the helix of the motif regions. The different annotation between these regions and the sarcin-ricin motif, which has five base pairs in the stack, disallows STAR3D to make the correct prediction.

### Structural alignments of homologous rRNAs

We also tested the performance of STAR3D on aligning the 3D structures of homologous 16S and 23S rRNAs. The benchmarking dataset includes three 23S rRNA chains from three different species: *H. marismortui* (PDB 1s72, chain 0), *Escherichia coli* (*E. coli*; PDB 2aw4, chain B) and *Thermus thermophilus* (*T. thermophilus*; PDB 2j01, chain A); and two 16S rRNA chains from two species: *T. thermophilus* (PDB 2avy, chain A) and *E. coli* (PDB 1j5e, chain A). We also used the two manually generated alignments of these 16S rRNAs as references. The first one is the Crystallographer alignment, which is implied in the numbering system used by the crystallographers; the second one is the Composite alignment, which is hand-crafted and based on comparative analysis. They have been used as benchmarking dataset before (15,20) (note that no such alignments are available for 23S rRNAs). R3D Align is also included in this benchmarking. All the tools are installed locally on a DELL XPS Desktop with Intel i7-4770 CPU at 3.40 GHz with 16 GB of RAM. To make the comparison fair, only one thread is allowed in the experiments.

First, we compare the running time of five tools for the rRNA alignments (see Table 2). It can be seen that STAR3D improves the time efficiency of the other tools by ten to a thousand folds. The adoption of the MaxSub algorithm (50) to refine the original 3D structural alignments may cause the huge time consumption in SARA. For STAR3D, the major running time reduction comes from the computation of loop alignments. Assuming the total lengths of loop regions for two RNA sequences are *n*_1_ and *n*_2_, the time complexity of loop alignment is *O*(*n*_1_ × *n*_2_). In STAR3D, one loop region only needs to be compared with another one marked by the *e*-stacks. Thus the time complexity is *O*((*n*_1_ × *n*_2_)/*m*), where *m* denotes the number of *e*-stack pairs in the consensus. With the relatively large number of stacks in RNAs with complex structures, our method can significantly improve the efficiency of the loop alignments.

Based on manually generated alignments of two 16S rRNAs, the accuracy of five tools is also examined. The results are shown in Table 3. It can be seen that both R3D Align and STAR3D achieve the maximum true positive number if the background dataset is the Composite dataset. The accuracy of STAR3D is slightly lower than R3D Align because it detects more nucleotide matches in the two sequences. However, for the Crystallographer dataset, STAR3D outperforms the other three tools. So STAR3D is not only highly efficient but also an accurate algorithm when it is used to align large homologous RNA molecules.

**Table 3.** Summary of alignments between the *T. thermophilus* and *E. coli* 16S RNAs (PDB: 1j5e, chain A and PDB: 2avy, chain A)

	Manual		3D structural alignment				
	Crystallographer	Composite	ARTS	LaJolla	SARA	R3D Align	STAR3D
Number of aligned nucleotides	1488	1414	1116	1106	1343	1400	1466
Agreeing with Composite	1401	1414	1056	1101	1240	**1362**	**1362**
Agreeing with Crystallographer	1488	1401	1081	1030	1276	1354	**1414**

Best performance is set to bold.

## DISCUSSION

In this article, we have proposed a novel tool, named STAR3D, for RNA 3D structural alignment. First it detects the conserved double-stranded regions in two input RNAs by joining the matches of small stack components. Then the consensus of stacks is assembled based on the 3D structural similarity and 2D compatible relationship. Its underlying tree-like topology leads to the ordering of loop regions. In addition, the rigid transformation of the aligned stacks can guide the 3D alignment of the loop regions. As a result, each loop only needs to be compared with its partner in the other sequence by using the superimposition of the conserved stacks. Finally, we combine the stack alignment and all the loop alignments as the final result. This ‘two-step’ strategy is derived on the basis of three observations. First, insertions and deletions are rarely seen in the conserved helical regions, which means that the ungapped extension is applicable to the stacks; second, the 3D structural similarity of conserved stacks is higher than that of random stacks; third, the stack regions are easier to annotate, even for low resolution PDB structures, so the stack alignment can be used as an anchor for the loop alignment. By integrating these properties into the design, STAR3D avoids the complex computation of secondary structure comparison. Furthermore, the one-to-one loop alignments, which replace the all-to-all base matching in entire single-stranded regions, reduce the running time of STAR3D for large RNAs significantly. The benchmark results show that the prediction accuracy of STAR3D outperforms the state-of-the-art tools, and does so with higher efficiency. What's more, STAR3D can be easily implemented with multi-thread support. The detection of *e*-stack pairs depends on ungapped alignment. The computation at each diagonal can be performed at an individual thread. For maximum clique finding, the Bron–Kerbosch algorithm can be implemented in parallel too. In the last step, the alignments of loop regions are independent, and can be deployed in different threads as well.

A potential expansion of STAR3D is to implement a local alignment version of the tool. From the experiment of aligning the GNRA motif and the 23S rRNA, it can be seen that STAR3D is sensitive to local similarities in RNA 3D structures. On the other hand, it is natural to convert STAR3D into finding local alignments. In the original method, only the maximum clique in the compatible graph is chosen to build the structural tree. To develop a local alignment approach, we can change STAR3D to deal with multiple cliques. For each one, a local alignment can be generated by only comparing the loops covered by the aligned stacks. With this new method, we anticipate that more structural motifs will be found in the functional ncRNAs.

Another direction for future study is to incorporate comparative methods into STAR3D. There are two approaches. The first one is to use the comparative methods to improve the alignments of the RNAs with low resolution coordinates. The excessive flexibility of atomic positions challenges the prediction of base pairing interactions, which may affect the performance of STAR3D. To solve the problem, we plan to design an iterative pipeline to find the base pairs in the low resolution RNA structures. First, we need a homology of the target that has high resolution 3D structural data. Hence a better annotation of base pairs for the target can be inferred by aligning two RNAs with STAR3D. In the following run, these predicted base pairs can be used as the secondary structural information in STAR3D to generate a more precise alignment. This procedure is continued until no new base pairs can be detected for the target. Considering the high efficiency of STAR3D, the time consumption of the pipeline should be practical. In addition, the low resolution RNAs can be aligned to other RNAs more accurately with the inferred base pairs. The second way is to find the 3D structural conservation among the RNAs in one family by using comparative methods. A hierarchical clustering based method, which is similar to CLUSTALW (51), is adopted. A 3D consensus structure of two RNAs can be constructed by connecting the centroids of the mapped nucleotides. Then, by merging the sub-clusters we can find the consensus for the whole family and its corresponding multiple sequence alignment.

## AVAILABILITY

http://genome.ucf.edu/STAR3D.
